# Personality profiles and usage experience are associated with trust and dependence on generative AI: a latent profile analysis

**DOI:** 10.3389/fpsyg.2026.1810554

**Published:** 2026-06-18

**Authors:** Mengru Zhang, Hang Zhao, Yunfei Wang

**Affiliations:** 1 Department of Industrial Design, Hongik University, Seoul, Republic of Korea; 2 School of Future Design, Beijing Normal University, Zhuhai, China

**Keywords:** big five personality traits, evolutionary mismatch, GAI dependence, GAI trust, GAI usage experience, latent profile analysis

## Abstract

**Introduction:**

Generative artificial intelligence (GAI) is increasingly integrated into daily decision-making and task performance, as users rely on it to improve productivity, support decision-making, and reduce perceived human error. However, GAI outputs may contain inaccuracies, misleading content, or algorithmic biases. These issues may contribute to uncritical acceptance or excessive dependence among users, potentially posing risks to individuals and third parties. From an individual perspective, this study systematically explores how personality traits and text-based GAI usage experience interact in relation to users’ trust in and dependence on text-based GAI.

**Methods:**

This study was based on a survey of 502 users measuring personality, usage experience, trust, dependence, self-efficacy, and critical thinking.

**Results:**

Significant interaction effects were found. Among high-experience users, the Well-Adjusted Type showed the highest dependence and self-efficacy, along with relatively high trust and critical thinking. The Outgoing-Unstructured Type showed the highest overall trust and, among high-experience users, the highest critical thinking, while its dependence did not differ significantly between low- and high-experience users. Overall, the Emotionally-Sensitive Type showed the lowest mean levels across the four outcomes, which may indicate a more cautious pattern of text-based GAI engagement.

**Discussion:**

These findings are discussed in relation to an evolutionary-cognitive perspective on how users may engage with human-like communicative cues in text-based GAI. The study emphasizes the importance of personalized text-based GAI usage strategies to mitigate misuse risks.

## 1 Introduction

Generative artificial intelligence (GAI) holds immense potential across numerous fields. It is built upon large language models (LLMs). It can produce highly coherent, logically consistent, and human-like text (Zhang Z. et al., 2025). Due to its low barrier to entry and broad applicability, GAI has rapidly gained popularity and become embedded in various aspects of learning, work, and daily life (Rapp et al., 2025). Billions of users now turn to GAI for tasks such as academic writing, poetry composition, software development, and contract drafting (Metz, 2022; Reed, 2022; Tung, 2023). This is also true in China and South Korea. Users there often access both global and locally developed text-based GAI tools. Examples include ChatGPT, Google Gemini, HyperCLOVA X, DeepSeek, Doubao, Kimi, and ERNIE Bot (Wenxin Yiyan). Some users treat GAI as a helpful tool to improve efficiency and output quality (Athar, 2025). However, recent studies suggest that some users trust GAI outputs without sufficient verification (Klingbeil et al., 2024; Sætra, 2023). Others may show excessive dependence on GAI by delegating entire tasks or substantial parts of tasks to it, or by accepting its suggestions without sufficient critical evaluation. Such patterns of misuse may be associated with risks such as harmful use, ethical concerns, and misinformation (Abbas et al., 2024).

User differences in GAI interactions go beyond frequency or task type. They may also be reflected in users’ trust in GAI outputs and their dependence on GAI during task execution (Choudhury and Shamszare, 2023). Research shows that GAI can quickly produce plausible responses. However, GAI outputs may contain errors or fabricated information, often termed “hallucinations” (Shen et al., 2023). Notably, these inaccuracies are frequently presented persuasively, with GAI sometimes providing seemingly rational justifications in follow-up responses, further reinforcing their superficial credibility (Dwivedi et al., 2023). Unverified use of GAI outputs may increase misuse risks. It may also expose users to biased or misleading content, increasing the risk of biased judgments or misleading interpretations. Despite some GAI systems cautioning users to verify outputs, many still place excessive trust in GAI and accept GAI outputs uncritically (Athar, 2025). Concurrently, a growing number of users show high dependence on GAI, struggling to complete tasks or make decisions without GAI assistance (Candrian and Scherer, 2022; Choudhury and Shamszare, 2023; Klingbeil et al., 2024). However, not all users display excessive trust or excessive dependence. Some leverage their expertise and critical evaluation skills to selectively adopt GAI outputs, using GAI as a tool to boost efficiency and deepen knowledge exploration (Athar, 2025). Others refine GAI outputs through secondary creation, which may lead to better outcomes than working alone (Zhou and Sterman, 2024). Thus, user differences in GAI adoption are closely tied to trust and dependence levels.

Trust in and dependence on GAI can be understood not only as patterns of technology use but also in relation to evolved social cognition. In human social life, cooperation and reciprocity offered benefits such as access to resources, protection, and future support, while also creating risks of cheating and non-reciprocation. These conditions may have favored cognitive mechanisms for social exchange, enabling individuals to recognize interaction partners, remember previous interactions, and assess whether others are likely to cooperate, reciprocate, or violate social exchange rules (Cosmides and Tooby, 1989). These social-exchange judgments also extend to human communication. In communicative interaction, people do not merely process the surface content of a response. Rather, they draw on shared intentionality, common ground, interaction goals, and communicative cues to infer others’ intentions, understanding, and cooperative orientation (Tomasello, 2008). From this perspective, trust in and dependence on GAI may involve not only evaluations of technical performance but also the activation of social-cognitive heuristics related to agency, credibility, competence, and cooperative intent.

This theoretical link is especially relevant to text-based GAI. Although such systems do not possess human-like mental states, they can generate fluent, responsive, and context-sensitive language. Users may therefore encounter them not only as functional tools, but also as adaptive systems that appear able to respond to user input in nuanced ways (Zhou and Zhang, 2024). Dennett’s concept of the intentional stance suggests that people may sometimes treat an entity as if it were a rational agent with beliefs, desires, and goals in order to explain or predict its behavior, without assuming that the entity actually possesses human-like mental states (Dennett, 1987). In this sense, text-based GAI may provide artificial cues of agency, competence, and responsiveness that partially activate social inference processes.

Recent work by Reinecke et al. (2025) is consistent with this interpretation. They argue that LLMs may be especially prone to anthropomorphism because their human-like language and conversational form can operate as agentive cues. These cues may encourage users to interpret GAI responses as more than generated text, as if the system showed agency or human-like mental states. Such interpretations may increase trust and, in some cases, over-trust, particularly when anthropomorphism makes possible inaccuracies or hallucinations less apparent. Anthropomorphic interpretation may also be relevant to dependence. Mazhar et al. (2026) found that perceived anthropomorphism was positively associated with GAI dependency, and that perceived empathy and autonomy were linked to stronger psychological and behavioral reliance on these systems. Taken together, these theoretical and empirical perspectives suggest that text-based GAI can be situated within an evolutionary mismatch framework (Lim et al., 2026): users encounter fluent and responsive language cues that resemble human communication, even though these cues do not indicate that the system has corresponding genuine intentions or mental states. This framing helps position the present study’s examination of how personality profiles and GAI usage experience are associated with users’ trust in and dependence on text-based GAI.

Against this background, the diverse behaviors users show when interacting with GAI may be partly linked to their underlying personality traits. Personality traits are related to preferences, cognitive styles, and decision-making (Choudhury and Shamszare, 2023; Kovbasiuk et al., 2024). As relatively stable psychological dispositions, personality traits are associated with individuals’ thought patterns, emotional responses, and behaviors, and have been widely examined as important predictors of technology adoption (Devaraj et al., 2008). In the emerging GAI field, understanding user personality is important. Personality is often examined using the Big Five model. This model can help inform human-AI interaction design and improve understanding of user acceptance of AI systems (Tripathi et al., 2022). Existing studies have begun to explore how personality traits relate to GAI usage behaviors and general attitudes (Kim et al., 2025; Stein et al., 2024). Yet, deeper psychological relationships—particularly how traits are differentially related to trust in and dependence on GAI—remain understudied.

GAI usage experience is also associated with behavior. Experienced users tend to perceive GAI outputs as more helpful, whereas novices may find them less useful (Topsakal, 2024). Prior research has examined usage frequency as a proxy for attitudes (De Winter et al., 2024), but how trust and dependence differ across levels of GAI usage experience remains insufficiently investigated. Therefore, combining GAI usage experience with personality profiles can offer a more detailed understanding of user-GAI dynamics. This can also inform the design of more effective human-AI systems.

### 1.1 Big five personality traits and GAI usage

Personality traits are relatively stable and enduring patterns in how people think, feel, and act (Blevins et al., 2022; Devaraj et al., 2008). They help explain and predict individual attitudes and behaviors in different situations. Among numerous theoretical models, the Big Five model of personality traits, also known as the Five-Factor Model (FFM), is one of the most widely used frameworks for studying individual differences due to its broad applicability and predictive validity (Kang et al., 2024). Costa and McCrae (1992) described the Five-Factor Model as a dimensional representation of personality structure, including five core dimensions: Openness to Experience, Conscientiousness, Extraversion, Agreeableness, and Neuroticism. Specifically, individuals scoring high in Openness are typically more interested in novelty and more willing to learn and explore new ideas; those high in Conscientiousness exhibit strong self-discipline, organized behavior, and effective impulse control; individuals high in Extraversion display greater self-confidence and are usually talkative and energetic; individuals high in Agreeableness are friendly, cooperative, and compassionate; whereas those high in Neuroticism tend to experience emotional instability and are prone to negative emotions such as anxiety and depression (Riedl, 2022). Together, these five dimensions offer a systematic way to understand individual differences in how people respond to technology.

Extensive research has shown that personality traits are associated with technology acceptance, usage behavior, and attitudes (Devaraj et al., 2008). This relationship is also evident in user interactions with GAI, where personality traits have been identified as important factors linked to user attitudes toward GAI and interaction behaviors (Kim et al., 2025; Schepman and Rodway, 2022). Regarding interaction behaviors, users with different personality traits appear to interact with GAI in different ways (Küper et al., 2025). For example, Sindermann et al. (2022) linked Extraversion to more frequent GAI use and Agreeableness to daily problem-solving, while low Conscientiousness or high Neuroticism was associated with a higher likelihood of misuse in academic tasks. Hu et al. (2025) further suggest that users high in Neuroticism may exhibit stronger compulsive use and addictive tendencies. At the attitudinal level, individuals high in Agreeableness and Conscientiousness generally hold more positive attitudes toward GAI, while highly neurotic individuals may be more susceptible to negative emotions (Stein et al., 2024). However, users high in Conscientiousness may also exhibit cautious or even negative attitudes due to their high demands for precision (Park and Woo, 2022; Schepman and Rodway, 2022). Existing studies have explored the relationship between personality and trust in AI (Thiebes et al., 2021). They have also examined links between specific traits (e.g., Neuroticism), and excessive dependence on GAI (Hu et al., 2025). However, research on how different personality profiles are associated with distinct patterns of trust and dependence remains limited. In increasingly complex GAI environments, users with different personality profiles may show different tendencies in trusting system judgments, accepting suggestions, or depending on GAI for task execution. Therefore, investigating how Big Five-based personality profiles are associated with variations in user trust and dependence may help inform personalized interventions and system design.

### 1.2 GAI usage experience and interaction patterns

As users continue to use GAI, their accumulated usage experience may gradually change their attitudes toward the system and their understanding of its capabilities (Horowitz et al., 2024). This perceptual change may occur across multiple dimensions. Novice users typically rate ChatGPT’s communicative competence lower than experienced users initially, but their evaluations improve markedly with repeated interactions. Even experienced users show ongoing shifts in their assessments over extended periods of use (Gessinger et al., 2025). These cognitive shifts may be linked to users’ growing practical skills and evaluative judgment developed through ongoing use, as well as to their trust and attitudes toward GAI (Burton et al., 2020). Trust and dependence can change over time through ongoing interactions. These changes may be linked to how often and how deeply users engage with GAI. They may also be linked to how users continue to check the reliability of GAI outputs and adjust their behavior (Glikson and Woolley, 2020; Kahr et al., 2024). Most current research focuses on comparing trust before and after use (Cabiddu et al., 2022). It pays less attention to how trust changes across different levels of GAI usage experience. It also overlooks how dependence appears under different use conditions. Therefore, it is important to study whether users’ trust calibration and dependence formation toward GAI outputs change systematically with accumulated GAI usage experience. Since experience can be partly measured by time-related usage, this study groups users based on their GAI usage experience.

### 1.3 The roles of trust and dependence in GAI use

Trust and dependence have become important variables for analyzing users’ attitudes and behavioral differences when interacting with GAI, and they have received increasing academic attention in recent years (Glikson and Woolley, 2020; Hu et al., 2021). Lee and See (2004) define trust as a user’s positive attitude toward an automated system. This attitude reflects the user‘s belief that the system can effectively support their goals in uncertain or risky situations. In the context of GAI, trust is further characterized as a user’s subjective evaluation of the accuracy and reliability of GAI outputs (Hu et al., 2021). However, GAI often relies on large-scale datasets. These datasets are not always carefully vetted. Therefore, its outputs may contain both accurate and fabricated information (Shen et al., 2023). This complexity may complicate users’ ability to discern authenticity, potentially leading to misjudgments. When users detect false or inaccurate information, their trust may decline quickly, which can be reflected in reduced or even discontinued system use (Omrani et al., 2022). Conversely, some users may overestimate GAI’s capabilities, show overly high trust, and neglect necessary verification. This may increase the risk of misuse, poor decision-making, and even severe consequences such as academic misconduct (Choudhury and Shamszare, 2023). To mitigate these issues, scholars have proposed enhancing system transparency and explainability to support more stable user trust (Burton et al., 2020; Sharan and Romano, 2020). Yet, these approaches may not fully account for individual differences among users, which may limit their effectiveness. In this context, recent research has increasingly turned attention to user-specific traits. Personality is an important factor among these traits. It is associated with trust. Ben-Ner and Halldorsson (2007) suggest that individuals high in agreeableness and extraversion but low in Conscientiousness generally exhibit greater trust propensity. However, whether the higher trust propensity reflected in these traits also applies to the specific context of GAI remains underexplored. Moreover, trust is not static; existing literature has largely overlooked its possible changes during prolonged user-GAI interactions. Thus, whether users with different personality profiles exhibit meaningful differences in trust levels after sustained GAI use—and how such differences are associated with their behavior—remains a central question for this study.

Dependence refers to users’ behavioral tendency to adopt or follow GAI recommendations in specific task contexts (Küper et al., 2025). Specifically, it reflects the extent to which users delegate decision-making and execution authority to AI systems to maximize potential gains (Candrian and Scherer, 2022). Studies suggest that many users perceive AI-driven decisions as more reliable, objective, and less prone to error than human judgment (Klingbeil et al., 2024), which may contribute to greater dependence on GAI in critical choices. However, excessive dependence may erode users’ independent judgment and decision-making skills, potentially affecting task performance. Full delegation of decisions to AI can also negatively impact stakeholders. For instance, in legal settings, AI recommendations may contain systemic biases, potentially resulting in unjust rulings with serious legal repercussions (Leib et al., 2025). Furthermore, when users cede decision-making responsibility to AI, their sense of moral accountability may weaken. This weakening may increase their susceptibility to unethical behavior. This is especially true when they face temptations or moral dilemmas (Leib et al., 2024).

Notably, user dependence on GAI extends beyond high-stakes domains, manifesting as habitual behavior in everyday tasks. Many users habitually delegate tasks to AI systems, reflecting habitual reliance on AI’s perceived competence rather than deliberate trust. For example, Zhang et al. (2024) found that over 70% of academic users struggle to complete tasks without AI assistance, reporting noticeable anxiety during system outages. Darvishi et al. (2024) also highlight users’ growing tendency toward strong dependence on AI for writing and learning tasks. This suggests that users perceive AI as increasingly important in daily activities (Stojanov et al., 2024). To curb over-reliance, interventions such as improving output accuracy, refining explanatory feedback, and enhancing human-AI decision alignment have been proposed (Bansal et al., 2021; Kahr et al., 2024; Lu and Yin, 2021). Yet, even with such measures, individual differences in dependence persist. Recent research thus explores how user-specific traits—particularly personality—are associated with dependence. Studies suggest that users with different personality traits may show different behavioral tendencies when interacting with GAI (De Winter, 2024). This contributes to the study of individual differences in human-AI collaboration. For terminological consistency, this paper uses dependence to emphasize users’ behavioral reliance on GAI systems.

Although trust and dependence are highly correlated, they are not deterministically linked; high trust does not necessarily entail greater behavioral dependence (Patton and Wickens, 2024). However, existing literature often conflates the two concepts, measuring them as a single dimension (Scharowski et al., 2022). This ambiguity may limit a deeper understanding of user-GAI interactions. Specifically, it blurs the distinction between psychological attitudes and behavioral outcomes. Trust reflects subjective attitude. Dependence reflects behavioral manifestation. To address this limitation, this study explicitly differentiates trust from dependence and investigates how personality profiles and GAI usage experience are associated with users’ trust levels and dependence tendencies, thereby clarifying differences in users’ cognitive and behavioral responses.

### 1.4 The present study and hypotheses

To examine differences in GAI trust and dependence, this study employs Latent Profile Analysis (LPA) to identify latent personality profiles based on Big Five personality traits. We examine the main and interaction effects of these LPA-derived personality profiles and GAI usage experience on trust, dependence, self-efficacy, and critical thinking, hypothesizing that:

*H*1: Users form distinct latent personality profiles based on the Big Five framework.

*H*2: The Well-Adjusted Type (e.g., high Openness/Agreeableness, low Neuroticism) will report higher trust, self-efficacy, and critical thinking, whereas the Emotionally-Sensitive Type will report lower levels.

*H*3: High-experience users will report higher levels of trust, dependence, self-efficacy, and critical thinking than low-experience users.

*H*4: A significant interaction between LPA-derived personality profiles and GAI usage experience will be observed in users’ trust and dependence patterns.

## 2 Materials and methods

### 2.1 Participants

Participants were recruited through both online and offline channels. Online participants completed the survey via Questionnaire Star (a widely used Chinese online survey platform), whereas offline recruitment was conducted in person at workplaces and university campuses. The target population consisted of members of the general public with prior experience using text-based GAI tools. During recruitment, participants were informed that the study focused on text-based GAI use and were asked to confirm whether they had prior experience using text-based GAI tools, such as ChatGPT, Google Gemini, DeepSeek, Doubao, Kimi, or other similar systems that generate written responses through natural language interaction. Those without prior experience using text-based GAI tools did not meet the participation criteria for this study and were therefore not included in the final valid sample. Ethical approval for the study was obtained from the authors’ university. Prior to participation, all individuals reviewed and agreed to an informed consent document outlining the study’s objectives and procedures.

A total of 536 participants from China and South Korea were recruited, yielding 502 valid responses (*N* = 502). The sample comprised 180 males and 322 females. Age distribution was as follows: 126 participants were aged ≤ 25 years, 259 were aged 26–35, 99 were aged 36–45, and 18 were aged ≥ 46. Regarding occupation, the sample included 172 students (including 32 South Korean students, ranging from undergraduate to doctoral level), 146 corporate employees (24 from South Korea), 76 education professionals (8 from South Korea), 59 freelancers (5 from South Korea), and 49 individuals in other occupations (3 from South Korea). Overall, the final sample comprised participants from China and South Korea with varied age and occupational backgrounds (Table 1).

**Table 1:** Descriptive statistics of participants.

Characteristics	Items	Number of people	Percentage
Sex	Male	180	35.90%
	Female	322	64.10%
Age	≤ 25 years old	126	25.10%
	26–35 years old	259	51.60%
	36–45 years old	99	19.70%
	≥ 46 years old	18	3.60%
Occupation	Student	172 (32)	34.3% (6.4%)
	Office worker	146 (24)	29.1% (4.8%)
	Educator	76 (8)	15.1% (1.6%)
	Freelancer	59 (5)	11.8% (1.0%)
	Other occupation	49 (3)	9.8% (0.6%)

The numbers in parentheses indicate the sample size of Korean participants.

### 2.2 Measures

#### 2.2.1 Mini-international personality item pool

This study employed the 20-item Mini-International Personality Item Pool (Mini-IPIP) to assess the Big Five personality traits based on the Five-Factor Model (Donnellan et al., 2006). Each dimension consisted of four items rated on a 5-point Likert scale (1 = strongly disagree to 5 = strongly agree). Subscale scores ranged from 4 to 20, with higher scores indicating greater trait intensity. Internal consistency in the present sample was satisfactory for all five dimensions: Openness (*α* = 0.87), Conscientiousness (α = 0.86), Extraversion (α = 0.84), Agreeableness (α = 0.82), and Neuroticism (α = 0.86).

#### 2.2.2 GAI usage experience

GAI usage experience was assessed through two author-developed items: (1) How long have you been using GAI? with response options: “Within 6 months” or “More than 6 months”; (2) On average, how many hours per week do you use GAI? with response options: “Less than 5 h,” “5–10 h,” “10–20 h,” and “More than 20 h.” For the main group comparison, participants were classified into high- and low-experience groups based on the weekly usage intensity item. Participants who reported using GAI for more than 10 h per week were classified as high-experience users, whereas those reporting 10 h or less per week were classified as low-experience users. The duration-of-use item was collected to describe participants’ usage background but was not used as the grouping criterion in the main analyses.

#### 2.2.3 Trust

Trust in GAI was measured using the situational trust scale proposed by Dolinek and Wintersberger (2022), which was adapted from Holthausen et al. (2020). To enhance its applicability across diverse contexts, two additional items were incorporated based on recommendations by Hoff and Bashir (2015). The final scale consisted of 8 items rated on a 7-point Likert scale (1 = strongly disagree, 7 = strongly agree), with items 2, 3, and 4 reverse-coded. Higher scores indicate greater trust in GAI. The scale demonstrated excellent internal consistency in this study (*α* = 0.93).

#### 2.2.4 Dependence

Users’ dependence on GAI was assessed using the Dependence Scale developed by Zhang X. et al. (2025). The scale consisted of 8 items rated on a 7-point Likert scale (1 = strongly disagree, 7 = strongly agree), with higher scores indicating stronger dependence on GAI. In the present study, the scale demonstrated excellent internal consistency reliability (α = 0.94).

#### 2.2.5 Self-efficacy

The General Self-Efficacy Scale for Generative AI (GSE-GAI) was used to assess participants’ self-reported self-efficacy in engaging with GAI technologies (Morales-García et al., 2024). The scale includes six items rated on a 4-point Likert scale (1 = completely incorrect, 4 = completely correct). Previous research has demonstrated its construct validity and satisfactory model fit (Woo et al., 2025). In the present study, the scale demonstrated good internal consistency (α = 0.87).

#### 2.2.6 Critical thinking

Critical thinking was measured using items adapted from Gerlich (2025), who modified the Halpern Critical Thinking Assessment (HCTA) and Terenzini’s self-reported measures of critical thinking development (Terenzini et al., 1995). The scale comprised 8 items across three dimensions: (1) Critical Evaluation and Reflection on Information Sources (4 items, α = 0.78; e.g., “How often do you critically evaluate the sources of information you encounter?”), (2) Confidence in Discernment of Misinformation (1 item; e.g., “How confident are you in your ability to discern fake news from legitimate news?”), and (3) Skepticism and Scrutiny of AI-Generated Information (3 items, α = 0.75; e.g., “I analyze the credibility of the author when reading news or information provided by AI tools”). These items were combined into a composite measure (α = 0.89). Participants responded using different 6-point scales for each dimension: a frequency scale (1 = never, 6 = always) for Dimension 1 (Items 1, 3, 4, 5), a confidence scale (1 = not at all confident, 6 = extremely confident) for Dimension 2 (Item 2), and an agreement scale (1 = strongly disagree, 6 = strongly agree) for Dimension 3 (Items 6, 7, 8). Higher total scores indicate stronger self-reported critical thinking in GAI use.

### 2.3 Procedure

A cross-sectional quantitative survey was administered through both online and offline channels using Questionnaire Star. After providing informed consent, participants were presented with information regarding the study purpose, confidentiality, and voluntary participation. The questionnaire consisted of four sections: demographic information (age and gender), GAI usage experience, the Big Five personality inventory, and scales assessing trust, dependence, self-efficacy, and critical thinking. At the beginning of the questionnaire, participants were asked to indicate which GAI tools they had used and to answer the GAI-related items based on their experience with text-based GAI interactions. Responses from participants who did not report prior experience with text-based GAI tools were not included in the final valid sample. On average, participants took approximately 11 min to complete the questionnaire. Data were collected over a two-week period in October 2025. Invalid or inattentive responses, such as those with abnormally short completion times, were removed during data cleaning, resulting in 502 valid cases for subsequent analysis.

### 2.4 Data analysis

To classify participants based on their Big Five personality traits, this study employed LPA, a person-centered approach that probabilistically assigns individuals to latent classes and reveals heterogeneity in response patterns and psychological attributes (Spurk et al., 2020). Models with two to four classes were estimated using Mplus 8.8. Model fit was evaluated using several criteria: Akaike Information Criterion (AIC), Bayesian Information Criterion (BIC), sample-size adjusted BIC (aBIC), Entropy, and the significance levels of the Lo–Mendell–Rubin (LMR) likelihood ratio test and the Bootstrap Likelihood Ratio Test (BLRT). As the number of classes increased, AIC, BIC, and aBIC values consistently decreased. The three-class solution exhibited optimal model fit (AIC = 6013.923; BIC = 6106.732; aBIC = 6036.902) and a high entropy value (0.980), indicating strong class separation. Both the LMR and BLRT tests were significant (*p* < 0.001), supporting the three-class model over the two-class solution. The estimated class probabilities were 0.129, 0.735, and 0.135; the class with the highest estimated probability represented the largest subgroup in the sample.

After identifying personality profiles, a 3 (Personality Profile: Emotionally-Sensitive Type, Well-Adjusted Type, Outgoing-Unstructured Type) × 2 (GAI Usage Experience: high vs. low) two-way ANOVA was conducted to examine the main and interaction effects on four dependent variables: trust, dependence, self-efficacy, and critical thinking. Levene’s tests for the full 3 × 2 ANOVA models were conducted for each dependent variable, and the results are reported in Table 2. When significant interaction effects were observed, simple effects analyses were conducted to further examine group differences. Given the unequal personality-profile group sizes and the significant full-model Levene’s test results, Games-Howell *post-hoc* tests were used consistently for pairwise comparisons among personality profiles. Independent-samples *t* tests were also conducted within each personality profile to compare the dependent variables between the low and high GAI usage experience groups. When equal variances were not assumed, Welch-corrected t tests were reported.

**Table 2:** Two-way ANOVA results for GAI dependence, trust, self-efficacy, and critical thinking.

Dependent variable	Source	SS	df	MS	F	*p*	ηp^2^	Levene’s F	*p*
Dependence	Personality type	102.816	2	51.408	33.286	< 0.001	0.118	53.385	< 0.001
	GAI usage experience	38.486	1	38.486	24.919	< 0.001	0.048		
	Personality Type × GAI usage experience	13.586	2	6.793	4.398	0.013	0.017		
Trust	Personality type	139.874	2	69.937	41.805	< 0.001	0.144	56.960	< 0.001
	GAI usage experience	31.221	1	31.221	18.662	< 0.001	0.036		
	Personality type × GAI usage experience	62.275	2	31.137	18.612	< 0.001	0.070		
Self-efficacy	Personality type	81.253	2	40.627	176.836	< 0.001	0.416	16.116	< 0.001
	GAI usage experience	6.573	1	6.573	28.612	< 0.001	0.055		
	Personality type × GAI usage experience	6.088	2	3.044	13.250	< 0.001	0.051		
Critical thinking	Personality type	38.232	2	19.116	21.389	< 0.001	0.079	19.237	< 0.001
	GAI usage experience	9.222	1	9.222	10.319	0.001	0.020		
	Personality type × GAI usage experience	36.407	2	18.203	20.369	< 0.001	0.076		

EST, Emotionally-Sensitive Type; WAT, Well-Adjusted Type; OUT, Outgoing-Unstructured Type. Levene’s tests refer to the homogeneity of error variances for the full 3 × 2 ANOVA model for each dependent variable; df = (5, 496) for all Levene’s tests.

## 3 Results

### 3.1 Personality classification based on LPA

**Figure 1:**
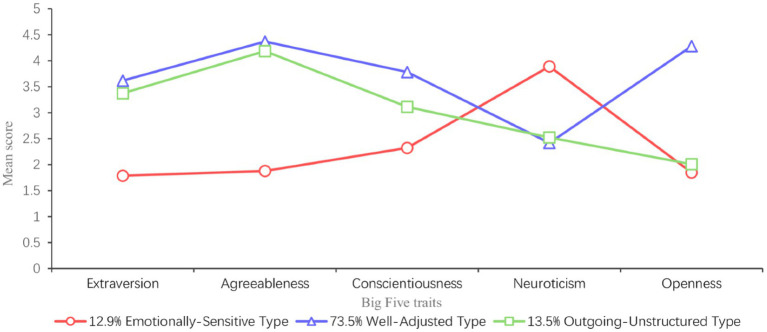
Latent profile classification of users based on the big five personality traits. Mean scores across extraversion, agreeableness, conscientiousness, neuroticism, and openness are shown for the three latent profiles identified via latent profile analysis (*N* = 502): emotionally-sensitive type (EST, 12.9%), well-adjusted type (WAT, 73.5%), and outgoing-unstructured type (OUT, 13.5%).

The first latent profile, labeled as the Emotionally-Sensitive Type (12.9%), was characterized by high levels of Neuroticism and low scores across the remaining four traits—Extraversion, Agreeableness, Conscientiousness, and Openness. This profile may reflect individuals with greater emotional sensitivity, lower sociability, and lower conscientiousness-related tendencies, suggesting a need for additional support in high-stakes or cognitively demanding interactions (Figure 1).

The second profile, designated as the Well-Adjusted Type (73.5%), showed generally higher scores on Extraversion, Agreeableness, Conscientiousness, and Openness, but low scores on Neuroticism. This pattern suggests relatively strong emotional stability, active social engagement, openness to new experiences, and a high degree of self-discipline. These characteristics may be associated with more reflective and adaptive patterns of GAI interaction.

The third profile, defined as the Outgoing-Unstructured Type (13.5%), displayed moderately high scores on Extraversion and Agreeableness, relatively lower scores on Openness and Neuroticism, and mid-range scores on Conscientiousness. While generally sociable and expressive, members of this profile may approach interactions in a more spontaneous and unstructured manner, with somewhat less conscientious deliberation than the Well-Adjusted Type.

### 3.2 Results of dependence

As shown in Table 2, the two-way ANOVA indicated a significant main effect of personality profile on GAI dependence, *F* (2, 496) = 33.286, *p* < 0.001, ηp^2^ = 0.118. This indicates that dependence differed significantly across personality profiles. The descriptive statistics for each profile were as follows: WAT (M = 4.895, SD = 1.044), OUT (M = 3.445, SD = 2.072), and EST (M = 3.263, SD = 2.034).

GAI usage experience also showed a significant main effect, *F* (1, 496) = 24.919, *p* < 0.001, ηp^2^ = 0.048. The mean dependence score was M = 5.171 (SD = 1.087) for the high GAI usage experience group and M = 3.589 (SD = 1.578) for the low GAI usage experience group.

The interaction between personality profile and GAI usage experience was also significant, *F* (2, 496) = 4.398, *p* = 0.013, ηp^2^ = 0.017. This indicates that the pattern of dependence across GAI usage experience levels may vary by personality profile.

As indicated by the full-model Levene’s test results reported in Table 2, variance homogeneity was not met for dependence across the six cells of the 3 × 2 ANOVA model. Therefore, Games-Howell *post-hoc* comparisons were conducted to examine differences among personality profiles. Within the low GAI usage experience group, EST reported significantly lower dependence than WAT (*p* = 0.047, 95% CI [−1.429, −0.008]). However, the difference between EST and OUT was not significant (*p* = 0.987), and the difference between WAT and OUT did not reach statistical significance (*p* = 0.051).

Within the high GAI usage experience group, EST reported significantly lower dependence than WAT (*p* = 0.024, 95% CI [−3.397, −0.244]), and WAT reported significantly higher dependence than OUT (*p* = 0.011, 95% CI [0.299, 2.456]). The difference between EST and OUT was not significant (*p* = 0.815). Detailed *post-hoc* results are presented in Table 3.

**Table 3:** Games-Howell *post-hoc* comparisons by outcome, GAI usage experience, and personality type.

Outcome variable	GAI usage experience	Personality type	*MD*	*SE*	*p*	95% CI	
						Lower	Upper
Dependence	Low	EST–WAT	−0.718^*^	0.296	0.047	−1.429	−0.008
		EST–OUT	0.064	0.420	0.987	−0.937	1.064
		WAT–OUT	0.782	0.324	0.051	−0.001	1.565
	High	EST–WAT	−1.820^*^	0.585	0.024	−3.397	−0.244
		EST–OUT	−0.443	0.724	0.815	−2.256	1.371
		WAT–OUT	1.378^*^	0.431	0.011	0.299	2.456
Trust	Low	EST–WAT	−0.254	0.301	0.676	−0.977	0.468
		EST–OUT	−1.443^**^	0.457	0.006	−2.534	−0.352
		WAT–OUT	−1.189^**^	0.367	0.006	−2.077	−0.301
	High	EST–WAT	−2.880^***^	0.351	< 0.001	−3.820	−1.940
		EST–OUT	−3.388^***^	0.467	< 0.001	−4.545	−2.230
		WAT–OUT	−0.508	0.319	0.268	−1.303	0.288
Self-efficacy	Low	EST–WAT	−1.155^***^	0.087	< 0.001	−1.362	−0.948
		EST–OUT	−1.309^***^	0.118	< 0.001	−1.590	−1.028
		WAT–OUT	−0.154	0.111	0.355	−0.420	0.113
	High	EST–WAT	−1.883^***^	0.070	< 0.001	−2.065	−1.701
		EST–OUT	−1.625^***^	0.128	< 0.001	−1.939	−1.310
		WAT–OUT	0.259	0.113	0.075	−0.022	0.540
Critical thinking	Low	EST–WAT	−0.447	0.213	0.097	−0.955	0.062
		EST–OUT	0.453	0.269	0.217	−0.188	1.094
		WAT–OUT	0.900^***^	0.215	< 0.001	0.385	1.414
	High	EST–WAT	−1.606^**^	0.352	0.002	−2.554	−0.659
		EST–OUT	−1.955^***^	0.400	< 0.001	−2.976	−0.934
		WAT–OUT	−0.349	0.198	0.204	−0.843	0.145

MD, mean difference; SE, standard error; CI, confidence interval; EST, Emotionally-Sensitive Type; WAT, Well-Adjusted Type; OUT, Outgoing-Unstructured Type. *p < 0.05; **p < 0.01; ***p < 0.001.

Independent-samples t tests were then conducted within each personality profile to compare dependence between the high and low GAI usage experience groups. Dependence did not differ significantly between GAI usage experience groups among EST users. Among WAT users, the high GAI usage experience group reported significantly higher dependence than the low GAI usage experience group, Welch’s t(174.351) = −14.358, *p* < 0.001. Among OUT users, the difference between the high and low GAI usage experience groups was not statistically significant. These results indicate that differences in dependence across GAI usage experience levels were mainly observed among WAT users, whereas EST and OUT users did not show significant differences between high and low GAI usage experience conditions. Detailed t test results are presented in Table 4.

**Table 4:** Independent-samples *t* test comparisons between low and high GAI usage experience groups within each personality type.

Outcome variable	Personality type	GAI usage experience	Mean	SD	Levene’s F	t	df	*p*	Cohen’s d
Dependence	EST	Low	3.200	2.051	0.008	−0.522	63	0.604	−0.167
		High	3.542	2.020					
	WAT	Low	3.919	0.995	7.124**	−14.358***	174.351	< 0.001	−1.812
		High	5.362	0.682					
	OUT	Low	3.137	2.015	0.226	−1.618	64	0.111	−0.414
		High	3.984	2.102					
Trust	EST	Low	3.091	2.091	17.261***	1.629	28.485	0.114	0.373
		High	2.358	1.199					
	WAT	Low	3.346	0.981	1.200	−18.478***	369	< 0.001	−2.051
		High	5.238	0.894					
	OUT	Low	4.535	2.306	24.828***	−2.552*	62.334	0.013	−0.587
		High	5.746	1.538					
Self-efficacy	EST	Low	1.445	0.491	0.234	−0.101	63	0.920	−0.032
		High	1.460	0.227					
	WAT	Low	2.600	0.607	83.438***	−12.348***	162.538	< 0.001	−1.615
		High	3.343	0.370					
	OUT	Low	2.754	0.625	0.767	−2.165*	64	0.034	−0.554
		High	3.085	0.541					
Critical thinking	EST	Low	3.479	1.380	2.025	1.758	63	0.084	0.562
		High	2.719	1.212					
	WAT	Low	3.926	1.068	41.311***	−3.773***	163.841	< 0.001	−0.491
		High	4.326	0.660					
	OUT	Low	3.026	1.238	1.160	−5.634***	64	< 0.001	−1.442
		High	4.675	0.951					

EST, Emotionally-Sensitive Type; WAT, Well-Adjusted Type; OUT, Outgoing-Unstructured Type. Welch-corrected t tests were reported when Levene’s test was significant. For Levene’s tests, df = (1, 63), (1, 369), and (1, 64) for EST, WAT, and OUT comparisons, respectively. *p < 0.05; **p < 0.01; ***p < 0.001.

**Figure 2:**
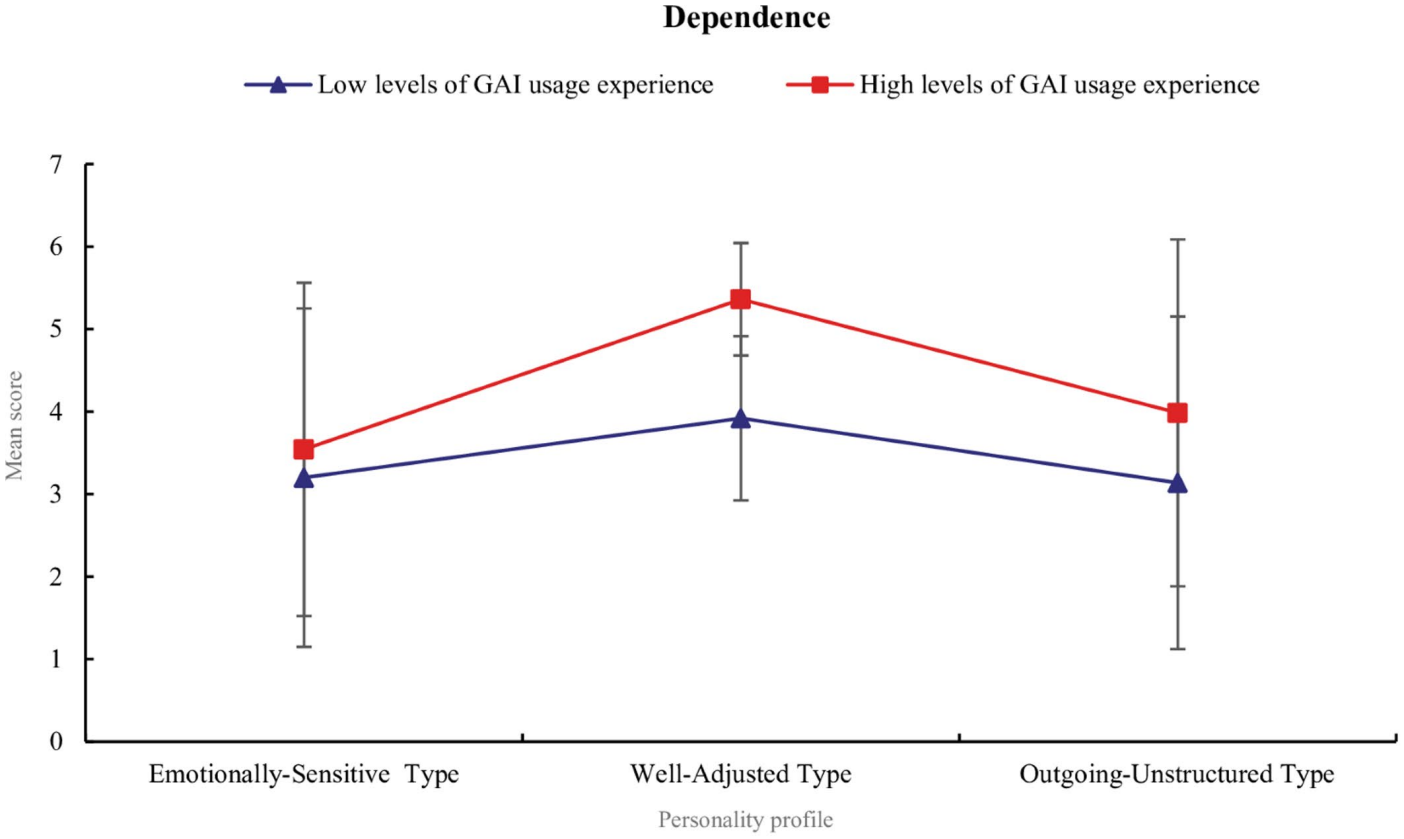
Interaction effect of personality profile and GAI usage experience on dependence. Mean dependence scores are shown for the three personality profiles (EST, WAT, OUT) across low and high GAI usage experience groups. Error bars represent ±1 standard deviation.

Notably, the Well-Adjusted Type showed the highest mean dependence scores across both usage experience groups, and this descriptive pattern was more pronounced among high-experience users (Figure 2).

### 3.3 Results of trust

As shown in Table 2, the two-way ANOVA indicated a significant main effect of personality profile on trust in GAI, *F* (2, 496) = 41.805, *p* < 0.001, ηp^2^ = 0.144. This indicates that trust differed significantly across personality profiles. The descriptive statistics for each profile were as follows: WAT (M = 4.626, SD = 1.279), OUT (M = 4.975, SD = 2.130), and EST (M = 2.956, SD = 1.970).

GAI usage experience also showed a significant main effect, *F* (1, 496) = 18.662, *p* < 0.001, ηp^2^ = 0.036. The mean trust score was M = 5.160 (SD = 1.144) for the high GAI usage experience group and M = 3.515 (SD = 1.697) for the low GAI usage experience group.

The interaction between personality profile and GAI usage experience was also significant, *F* (2, 496) = 18.612, *p* < 0.001, ηp^2^ = 0.070. This indicates that the pattern of trust across GAI usage experience levels may vary by personality profile.

As indicated by the full-model Levene’s test results reported in Table 2, variance homogeneity was not met for trust across the six cells of the 3 × 2 ANOVA model. Therefore, Games-Howell *post-hoc* comparisons were conducted to examine differences among personality profiles. Within the low GAI usage experience group, trust did not differ significantly between EST and WAT (*p* = 0.676). However, EST reported significantly lower trust than OUT (*p* = 0.006, 95% CI [−2.534, −0.352]), and WAT also reported significantly lower trust than OUT (p = 0.006, 95% CI [−2.077, −0.301]).

Within the high GAI usage experience group, EST reported significantly lower trust than both WAT (*p* < 0.001, 95% CI [−3.820, −1.940]) and OUT (*p* < 0.001, 95% CI [−4.545, −2.230]). The difference between WAT and OUT was not significant (*p* = 0.268). Detailed *post-hoc* results are presented in Table 3.

Independent-samples t tests were then conducted within each personality profile to compare trust between the high and low GAI usage experience groups. Trust did not differ significantly between GAI usage experience groups among EST users. Among WAT users, the high GAI usage experience group reported significantly higher trust than the low GAI usage experience group, t(369) = −18.478, *p* < 0.001. A similar pattern was observed among OUT users, with the high GAI usage experience group reporting significantly higher trust than the low GAI usage experience group, Welch’s t(62.334) = −2.552, *p* = 0.013. These results indicate that differences in trust across GAI usage experience levels were mainly observed among WAT and OUT users, whereas EST users did not show a significant difference between high and low GAI usage experience conditions. Detailed t test results are presented in Table 4.

**Figure 3:**
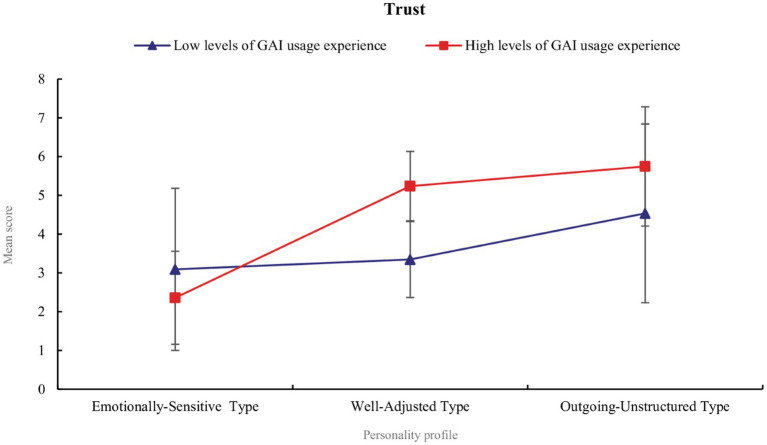
Interaction effect of personality profile and GAI usage experience on trust. Mean trust scores are shown for the three personality profiles (EST, WAT, OUT) across low and high GAI usage experience groups. Error bars represent ±1 standard deviation.

Notably, OUT users showed the highest mean trust scores across both GAI usage experience groups, although this descriptive advantage over WAT was relatively small in the high GAI usage experience group (Figure 3).

### 3.4 Results of self-efficacy

As shown in Table 2, the two-way ANOVA indicated a significant main effect of personality profile on self-efficacy, *F* (2, 496) = 176.836, *p* < 0.001, ηp^2^ = 0.416. This indicates that self-efficacy differed significantly across personality profiles. The descriptive statistics for each profile were as follows: WAT (M = 3.103, SD = 0.576), OUT (M = 2.874, SD = 0.613), and EST (M = 1.448, SD = 0.453).

GAI usage experience also showed a significant main effect, *F* (1, 496) = 28.612, *p* < 0.001, ηp^2^ = 0.055. The mean self-efficacy score was M = 3.243 (SD = 0.538) for the high GAI usage experience group and M = 2.346 (SD = 0.780) for the low GAI usage experience group.

The interaction between personality profile and GAI usage experience was also significant, *F* (2, 496) = 13.250, *p* < 0.001, ηp^2^ = 0.051. This indicates that the pattern of self-efficacy across GAI usage experience levels may vary by personality profile.

As indicated by the full-model Levene’s test results reported in Table 2, variance homogeneity was not met for self-efficacy across the six cells of the 3 × 2 ANOVA model. Therefore, Games-Howell *post-hoc* comparisons were conducted to examine differences among personality profiles. Within the low GAI usage experience group, EST reported significantly lower self-efficacy than both WAT (*p* < 0.001, 95% CI [−1.362, −0.948]) and OUT (p < 0.001, 95% CI [−1.590, −1.028]). The difference between WAT and OUT was not significant (*p* = 0.355).

Within the high GAI usage experience group, EST also reported significantly lower self-efficacy than both WAT (*p* < 0.001, 95% CI [−2.065, −1.701]) and OUT (*p* < 0.001, 95% CI [−1.939, −1.310]). The difference between WAT and OUT was not significant (*p* = 0.075). Detailed *post-hoc* results are presented in Table 3.

Independent-samples t tests were then conducted within each personality profile to compare self-efficacy between the high and low GAI usage experience groups. Self-efficacy did not differ significantly between GAI usage experience groups among EST users. Among WAT users, the high GAI usage experience group reported significantly higher self-efficacy than the low GAI usage experience group, Welch’s t(162.538) = −12.348, *p* < 0.001. A similar pattern was observed among OUT users, with the high GAI usage experience group reporting significantly higher self-efficacy than the low GAI usage experience group, t(64) = −2.165, *p* = 0.034. These results indicate that differences in self-efficacy across GAI usage experience levels were mainly observed among WAT and OUT users, whereas EST users did not show a significant difference between high and low GAI usage experience conditions. Detailed t test results are presented in Table 4.

**Figure 4:**
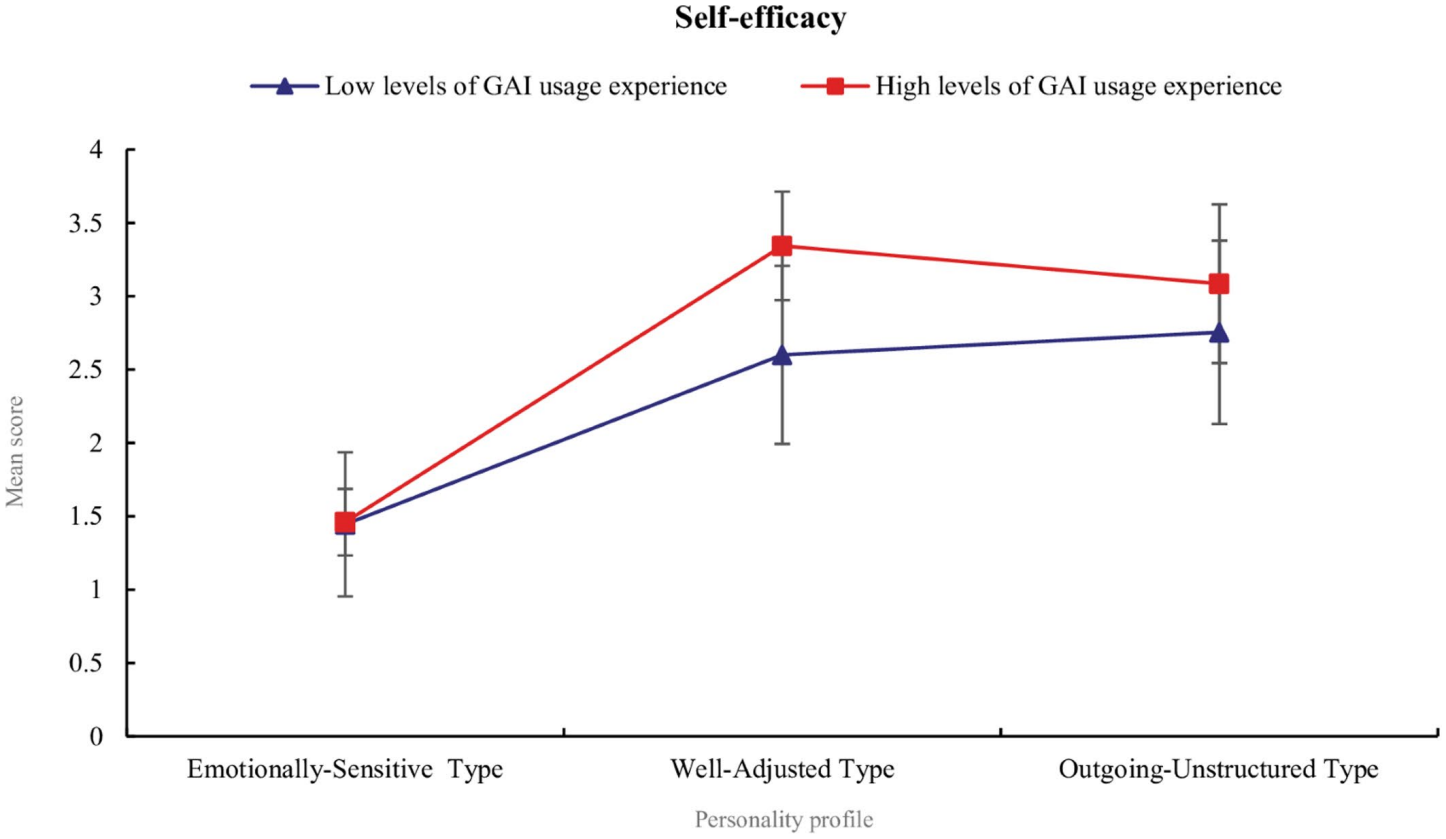
Interaction effect of personality profile and GAI usage experience on self-efficacy. Mean self-efficacy scores are shown for the three personality profiles (EST, WAT, OUT) across low and high GAI usage experience groups. Error bars represent ±1 standard deviation.

Notably, the descriptive pattern of self-efficacy differed across GAI usage experience groups: OUT users showed the highest mean self-efficacy scores in the low GAI usage experience group, whereas WAT users showed the highest mean scores in the high GAI usage experience group. EST users showed the lowest mean self-efficacy scores across both groups (Figure 4).

### 3.5 Results of critical thinking

As shown in Table 2, the two-way ANOVA indicated a significant main effect of personality profile on critical thinking, *F* (2, 496) = 21.389, *p* < 0.001, ηp^2^ = 0.079. This indicates that critical thinking differed significantly across personality profiles. The descriptive statistics for each profile were as follows: WAT (M = 4.196, SD = 0.834), OUT (M = 3.626, SD = 1.388), and EST (M = 3.339, SD = 1.374).

GAI usage experience also showed a significant main effect, *F* (1, 496) = 10.319, *p* = 0.001, ηp^2^ = 0.020. The mean critical thinking score was M = 4.288 (SD = 0.792) for the high GAI usage experience group and M = 3.640 (SD = 1.231) for the low GAI usage experience group.

The interaction between personality profile and GAI usage experience was also significant, F (2, 496) = 20.369, *p* < 0.001, ηp^2^ = 0.076. This indicates that the pattern of critical thinking across GAI usage experience levels may vary by personality profile.

As indicated by the full-model Levene’s test results reported in Table 2, variance homogeneity was not met for critical thinking across the six cells of the 3 × 2 ANOVA model. Therefore, Games-Howell *post-hoc* comparisons were conducted to examine differences among personality profiles. Within the low GAI usage experience group, critical thinking did not differ significantly between EST and WAT (*p* = 0.097), or between EST and OUT (*p* = 0.217). However, WAT reported significantly higher critical thinking than OUT (*p* < 0.001, 95% CI [0.385, 1.414]).

Within the high GAI usage experience group, EST reported significantly lower critical thinking than both WAT (*p* = 0.002, 95% CI [−2.554, −0.659]) and OUT (*p* < 0.001, 95% CI [−2.976, −0.934]). The difference between WAT and OUT was not significant (*p* = 0.204). Detailed *post-hoc* results are presented in Table 3.

Independent-samples t tests were then conducted within each personality profile to compare critical thinking between the high and low GAI usage experience groups. Critical thinking did not differ significantly between GAI usage experience groups among EST users. Among WAT users, the high GAI usage experience group reported significantly higher critical thinking than the low GAI usage experience group, Welch’s t(163.841) = −3.773, *p* < 0.001. A similar pattern was observed among OUT users, with the high GAI usage experience group reporting significantly higher critical thinking than the low GAI usage experience group, t(64) = −5.634, *p* < 0.001. These results indicate that differences in critical thinking across GAI usage experience levels were mainly observed among WAT and OUT users, whereas EST users did not show a significant difference between high and low GAI usage experience conditions. Detailed t test results are presented in Table 4.

**Figure 5:**
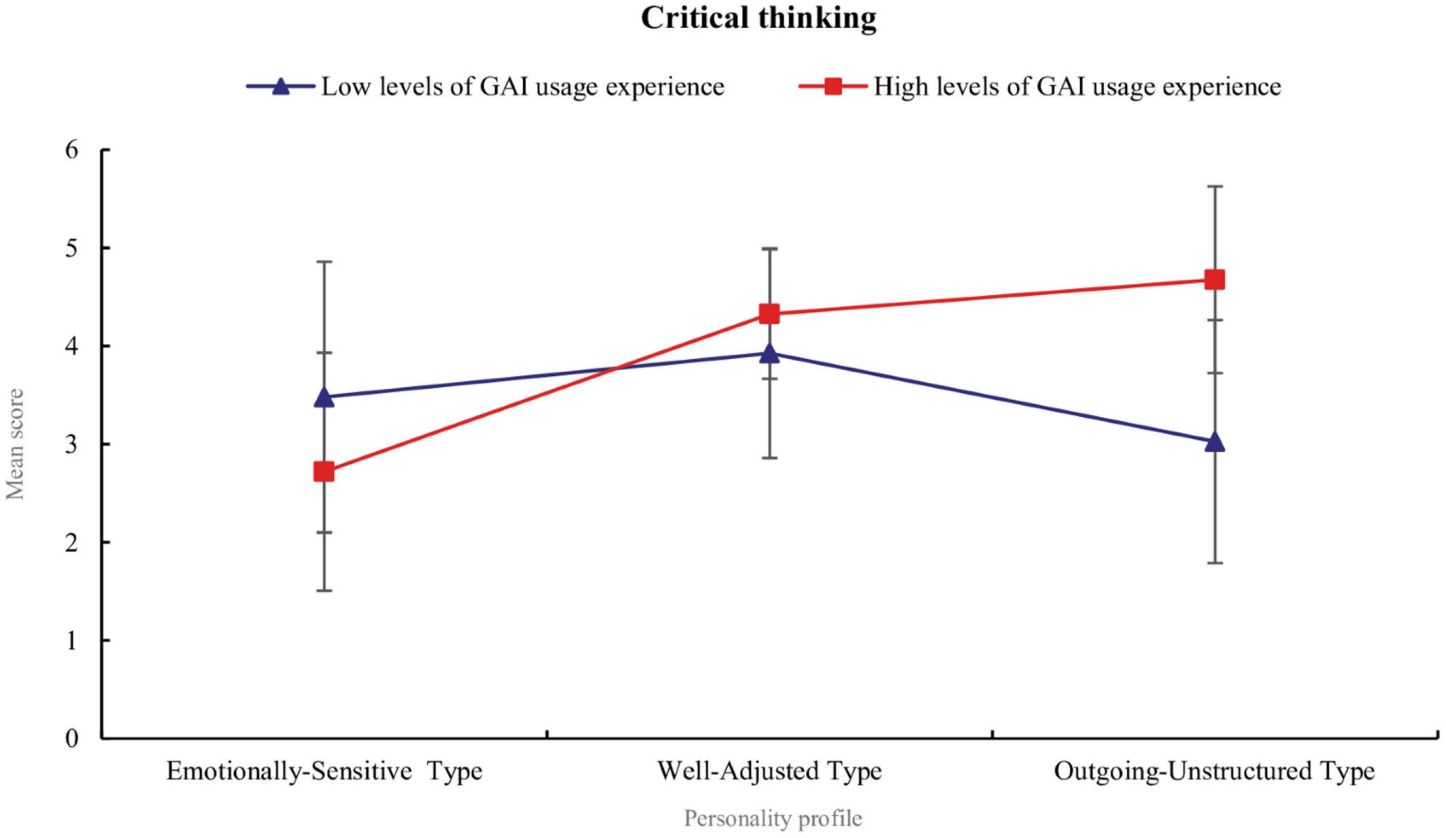
Interaction effect of personality profile and GAI usage experience on critical thinking. Mean critical thinking scores are shown for the three personality profiles (EST, WAT, OUT) across low and high GAI usage experience groups. Error bars represent ±1 standard deviation.

Notably, the descriptive pattern of critical thinking differed across GAI usage experience groups: WAT users showed the highest mean critical thinking scores in the low GAI usage experience group, whereas OUT users showed the highest mean scores in the high GAI usage experience group (Figure 5).

Summary of Hypothesis Testing: Overall, the identification of three distinct latent personality profiles supports H1. The significant main effects of GAI usage experience across trust, dependence, self-efficacy, and critical thinking support H3. The significant interaction effects between personality profiles and GAI usage experience on trust and dependence further support H4. H2 was partially supported: the Well-Adjusted Type (WAT) showed the expected advantage in overall mean self-efficacy and critical thinking, but not in trust, where the Outgoing-Unstructured Type (OUT) reported the highest overall mean scores. The Emotionally-Sensitive Type (EST) showed the lowest overall mean levels across all four outcomes, consistent with H2.

## 4 Discussion

This study examined the main and interaction effects of personality profiles and GAI usage experience on user engagement with text-based GAI. Overall, our empirical findings were generally consistent with our theoretical framework, while revealing several nuanced patterns. Hypotheses H1, H3, and H4 were supported, indicating the existence of distinct latent personality profiles, significant experience-related differences across the four outcomes, and significant personality profile × GAI usage experience interactions in trust and dependence. Notably, H2 was only partially supported: WAT showed the highest overall mean self-efficacy and the highest overall mean critical thinking score, whereas OUT showed the highest overall mean trust score and the highest mean critical thinking scores among high-experience users. Thus, the expected advantage of WAT was not consistent across all outcome variables. The following sections discuss these patterns and their possible psychological interpretations, with particular attention to how users may respond differently to the social cues provided by text-based GAI.

Seen in light of the theoretical framing introduced in the Introduction, the trust and dependence patterns observed here may partly reflect users’ different responses to the linguistic and social cues provided by text-based GAI. Although this study focused on text-based GAI rather than embodied agents, language itself may still have social-cognitive effects. Prior work on language-focused anthropomorphism suggests that verbal cues in conversational agents can shape perceived trust even in the absence of physical or visual embodiment (Brunswicker et al., 2025). At the same time, fluent and responsive language may also encourage users to perceive agency or human-like mental states in LLMs (Reinecke et al., 2025). From this perspective, text-based GAI can be understood as a possible mismatch-like or “supernormal” communicative cue: it presents signals that resemble human communication and may make the system appear more trustworthy; however, unlike interpersonal interaction, these signals do not mean that the system has corresponding intentions, understanding, or mental states. This interpretation is consistent with an evolutionary mismatch framework, which considers how evolutionarily novel AI-related cues may interact with evolved psychological mechanisms (Lim et al., 2026). The significant personality profile × GAI usage experience interactions in trust and dependence suggest that users’ responses to these cues were not uniform, but varied across personality profiles and usage experience levels. However, this interpretation should remain tentative, because the present study did not directly measure evolved cognitive mechanisms, anthropomorphism, or mind attribution.

### 4.1 Personality-based variations in GAI trust and dependence

The Emotionally-Sensitive Type (EST) showed a relatively cautious pattern of GAI engagement, as reflected in the lowest overall mean levels of trust, dependence, self-efficacy, and critical thinking. This pattern may be related to the profile’s elevated Neuroticism, together with its lower Openness and Extraversion. Empirical evidence suggests that individuals high in Neuroticism often show heightened anxiety and persistent insecurity when encountering novel technologies (Nilsen et al., 2024; Sharan and Romano, 2020), which may reduce their willingness to actively explore new technologies. This interpretation is consistent with Sharan and Romano (2020) conclusions regarding barriers to technology adoption. Hu et al. (2025) offer a different interpretation, suggesting that individuals high in Neuroticism may develop greater dependence on GAI as a way of regulating anxiety. However, the present findings did not support this pattern. This discrepancy may be partly related to the lower Openness and Extraversion observed in the present sample, as these traits may be associated with less proactive exploration and experimentation with GAI, helping to explain EST users’ more cautious engagement pattern. From the broader social-cognitive perspective introduced above, this cautious pattern may also be consistent with heightened vigilance or stronger error-avoidance tendencies, which can be understood as a possible uncertainty-management response to socially suggestive but nonhuman language cues. When text-based GAI provides cues that resemble human communication, EST users may be less inclined to treat them as reliable signs of competence, credibility, or cooperative intent.

The Well-Adjusted Type (WAT) showed a distinctive pattern of GAI engagement, characterized by relatively high trust and the highest overall mean levels of dependence, self-efficacy, and critical thinking. This overall pattern may be related to WAT users’ personality configuration, particularly their high Agreeableness and Openness combined with low Neuroticism. The combination of high Agreeableness and Openness may be associated with a receptive yet discerning attitude toward GAI systems (Park and Woo, 2022), which may in turn be related to more positive trust-related experiences. Related patterns have been discussed by Bawack et al. (2021) and Aliasghari et al. (2021). Importantly, their low Neuroticism may be associated with stronger emotional regulation and more deliberate decision-making when interacting with GAI systems. Taken together, the coexistence of elevated dependence with relatively high self-efficacy and critical thinking suggests that WAT users’ dependence may be embedded in a more active and confident form of GAI engagement. In theoretical terms, this pattern may reflect a more regulated response to GAI’s social and informational cues, in which users may be willing to draw on GAI support while still perceiving themselves as capable of evaluating its outputs. However, because these variables were assessed through self-report and the study used a cross-sectional design, this pattern should not be interpreted as evidence that WAT users’ higher dependence is necessarily adaptive or objectively appropriate.

The Outgoing-Unstructured Type (OUT) also showed a distinctive pattern of GAI engagement. OUT users reported the highest overall mean trust score, while their overall mean levels of dependence, self-efficacy, and critical thinking were higher than those of EST but lower than those of WAT. This pattern may be related to OUT users’ personality configuration, characterized by relatively high Extraversion and Agreeableness, lower Openness, and mid-range Conscientiousness. Relatively high Extraversion and Agreeableness may be associated with a more positive and receptive attitude toward GAI, which may help account for their higher trust levels. From a social-cognitive perspective, this high-trust pattern may also suggest greater receptiveness to socially engaging or readily accessible informational cues provided by GAI. At the same time, lower Openness and mid-range Conscientiousness may suggest that although OUT users are willing to use GAI, their judgment and verification of GAI outputs may be less systematic and deliberative than those of WAT users. Overall, OUT users may represent a high-trust but relatively less systematic pattern of GAI engagement.

### 4.2 Experience-related differences in GAI engagement

The empirical analysis showed significant experience-related differences in users’ trust in GAI, dependence, self-efficacy, and critical thinking. This finding is consistent with prior research (Gessinger et al., 2025). Specifically, high-experience users reported significantly higher levels than low-experience users across all four variables. These differences may be related not only to basic interface familiarity, but also to greater cognitive familiarity and accumulated experience with GAI systems. In the context of text-based GAI, repeated interaction may also give users more opportunities to interpret and test the linguistic and social cues provided by these systems, and to recalibrate their trust and dependence accordingly. Users with greater GAI usage experience may have a more nuanced understanding of GAI’s capabilities and limitations, including hallucinations and biases, as well as appropriate application contexts. Moreover, sustained interaction may be associated with greater operational confidence and self-efficacy, such that users may feel more capable of identifying and responding to irregularities in GAI outputs. Accordingly, the higher dependence reported by high-experience users may reflect a more experience-based tendency toward GAI use. High-experience users may be more willing to draw on GAI support while also feeling more capable of judging when to use it, how to use it, and when its outputs require further checking.

By contrast, low-experience users may have less developed mental models of GAI systems, possibly related to limited practice and feedback. This may be associated with greater uncertainty in evaluating GAI outputs and less stable trust–dependence patterns. From the social-cognitive perspective discussed above, and in line with the role of mental model development, limited experience may make it more difficult for users to judge whether GAI’s fluent and responsive language is primarily a communicative cue or a reliable indication of task-relevant competence and accuracy. In this context, low-experience users may have greater difficulty deciding when to accept, question, or reject GAI outputs. These findings suggest that experiential learning may be relevant to responsible GAI engagement. Future work could examine personalized training interventions that support novice users’ mental model development and may help promote safer and more effective human-AI collaboration by strengthening system literacy and critical thinking.

### 4.3 Interactive effects of personality and GAI usage experience

The investigation of the personality profile × GAI usage experience interactions revealed different patterns of GAI engagement across personality profiles. These interaction patterns suggest that GAI usage experience was not associated with trust and dependence in the same way across personality profiles. In other words, users with different personality profiles may differ in how they interpret and respond to the human-like language cues provided by text-based GAI. For Emotionally-Sensitive Type (EST) users, the high- and low-experience groups did not differ significantly in trust, dependence, self-efficacy, or critical thinking. This pattern may suggest that EST users showed a relatively stable cautious engagement style across different levels of GAI usage experience. Their personality configuration, characterized by elevated Neuroticism and lower Openness, may be associated with persistent uncertainty or hesitation when interacting with GAI systems. Taken together, these findings suggest that differences in GAI usage experience were not strongly associated with differences in EST users’ trust–dependence patterns in this sample.

For Well-Adjusted Type (WAT) users, the high-experience group reported significantly higher trust, dependence, self-efficacy, and critical thinking than the low-experience group. This pattern suggests that, among WAT users, greater usage experience may be associated with a broader pattern of GAI engagement, reflected not only in higher trust, self-efficacy, and critical thinking, but also in higher GAI dependence. In light of WAT users’ lower Neuroticism and higher Openness, this result may point to a relatively positive and stable pattern of GAI interaction among WAT users with greater accumulated experience. In theoretical terms, this pattern may be interpreted as a form of trust calibration through repeated interaction: among WAT users, greater GAI usage experience was associated with higher trust and dependence, while also being accompanied by higher self-efficacy and critical thinking. At the same time, the higher dependence observed among high-experience WAT users indicates that this experience-related pattern was not limited to greater confidence or evaluative engagement, but also involved stronger self-reported dependence on GAI.

For Outgoing-Unstructured Type (OUT) users, the high-experience group reported significantly higher trust, self-efficacy, and critical thinking than the low-experience group, whereas no significant difference in dependence was observed between the two groups. This pattern suggests that, among OUT users, greater GAI usage experience may be associated mainly with higher trust, self-efficacy, and critical thinking, rather than with a comparable difference in dependence. Unlike WAT users, for whom the high-experience group reported significantly higher trust, dependence, self-efficacy, and critical thinking than the low-experience group, OUT users showed a more selective experience-related pattern. In other words, among OUT users, greater GAI usage experience was associated with higher trust in GAI and higher self-reported self-efficacy and critical thinking, but not with a significant difference in GAI dependence. This selective pattern may suggest that high-experience OUT users were more receptive to GAI’s human-like language cues and informational support, but this greater receptiveness did not necessarily correspond to higher dependence.

### 4.4 Limitations and future research

Although this study offers insights into users’ psychological responses in human–AI interaction, several methodological limitations should be acknowledged.

First, this study used a cross-sectional survey design, which precludes causal conclusions about the relationships among personality traits, GAI usage experience, and users’ trust–dependence patterns. Future research could use longitudinal or experimental designs to examine whether and how these patterns vary over time.

Second, the main outcome variables were measured using self-report questionnaire scales. This may introduce social desirability bias and common method bias. This is especially relevant to dependence, self-efficacy, and critical thinking, as participants may overestimate their usage behavior, perceived ability, or evaluative rigor when using GAI. Therefore, these scores should be interpreted as self-reported perceptions rather than demonstrated behavior or competence. Procedural remedies were adopted to reduce these biases. Future research could incorporate objective behavioral data, such as system usage logs, prompt records, task performance, error-identification tasks, or behavioral indicators of dependence, to better assess users’ actual GAI use. In addition, although the Discussion situates the findings within an evolutionary-cognitive framework, the present study did not directly measure evolved cognitive mechanisms, anthropomorphism, or mind attribution. Therefore, these theoretical interpretations should be treated as tentative and should be examined more directly in future research.

Third, GAI usage experience was measured using author-developed items and divided into high- and low-experience groups based on predefined cutoffs. In the main analyses, this grouping was based on weekly usage intensity rather than duration of use. Although this binary grouping helped compare users with different experience levels, it may simplify the continuous and multidimensional nature of actual GAI usage experience. In addition, weekly usage intensity may conceptually overlap with dependence, because more frequent use can partly reflect stronger behavioral reliance on GAI. This overlap should be considered when interpreting the association between GAI usage experience and dependence. Future research could use validated multi-item scales, continuous indicators, or behavioral usage data to capture usage frequency, usage duration, task diversity, and interaction depth more accurately.

Fourth, participants in this study were mainly from China and South Korea, and the demographic composition of the sample was not fully balanced. Therefore, caution is needed when generalizing the findings to other cultural contexts or broader GAI user populations. Future research could further examine whether these LPA-derived personality profiles and trust–dependence patterns are also observed in Western or more culturally diverse samples.

Finally, this study focused exclusively on text-based GAI. Multimodal GAI, including image and video generation, is becoming increasingly prevalent. Future research could further examine whether the trust and dependence patterns observed in this study remain consistent across different generative modalities.

## 5 Conclusion

This study examined how personality profiles and GAI usage experience are jointly associated with users’ GAI engagement patterns. The study offers theoretical and practical insights into individual differences in text-based GAI use. Three personality profiles were identified: Emotionally-Sensitive Type (EST), Well-Adjusted Type (WAT), and Outgoing-Unstructured Type (OUT). The findings suggest that users’ GAI engagement patterns differed not only across personality profiles, but also across levels of usage experience. Specifically, EST users showed a relatively cautious engagement pattern across experience levels; among WAT users, high-experience users reported higher trust, dependence, self-efficacy, and critical thinking than low-experience users; and among OUT users, high-experience users reported higher trust, self-efficacy, and critical thinking than low-experience users, whereas OUT users’ dependence did not differ significantly across experience levels. These results suggest that both personality profiles and usage experience should be considered when understanding users’ trust in and dependence on GAI. Together, these findings are consistent with the evolutionary-cognitive framing introduced in this paper, suggesting that users may differ in how they engage with the human-like communicative cues of text-based GAI.

These findings have implications for GAI system design, user training, and risk mitigation. Future research and practice could further consider differences in users’ psychological profiles and usage experience to provide more targeted support. For lower-experience or more cautious users, support could focus on building basic system familiarity and usage confidence. For more highly engaged users, guidance could emphasize reflective evaluation and appropriate checking of GAI outputs. By incorporating an individual-difference perspective into GAI design and education, future work may help support safer, more effective, and more context-sensitive human–AI interaction.

## Data Availability

The original contributions presented in the study are included in the article/Supplementary material, further inquiries can be directed to the corresponding author.
